# Dr. Francis W. Higgins

**Published:** 1904-01

**Authors:** 


Dr. Francis W. Higgins, of Cortland, N. Y., dropped dead in his office December 18, 1903, aged 46 years. He graduated in medicine from the medical department of New York University in 1881. Dr. Higgins was a prominent physician in his region of the State and was a member of several medical societies. He attended the last meeting of the Medical Association of Central New York, where he read a paper which appears in this issue of the Journal. His sudden death in the prime of life is lamented by professional colleagues, friends and patients.

